# A new paradigm for antiangiogenic therapy through controlled release of bevacizumab from PLGA nanoparticles

**DOI:** 10.1038/s41598-017-03959-4

**Published:** 2017-06-16

**Authors:** Flávia Sousa, Andrea Cruz, Pedro Fonte, Inês Mendes Pinto, Maria Teresa Neves-Petersen, Bruno Sarmento

**Affiliations:** 1 i3S - Instituto de Investigação e Inovação em Saúde, Universidade do Porto, Rua Alfredo Allen 208, 4200-393 Porto, Portugal; 2 INEB - Instituto Nacional de Engenharia Biomédica, Universidade do Porto, Rua Alfredo Allen 208, 4200-393 Porto, Portugal; 3 ICBAS - Instituto Ciências Biomédicas Abel Salazar, Universidade do Porto, 4150-180 Porto, Portugal; 4 CESPU - Instituto de Investigação e Formação Avançada em Ciências e Tecnologias da Saúde, Rua Central de Gandra 1317, 4585-116 Gandra, Portugal; 5 INL, International Iberian Nanotechnology Laboratory, Avenida Mestre José Veiga, 4715-330 Braga, Portugal; 6 UCIBIO, REQUIMTE, Department of Chemical Sciences – Applied Chemistry Lab, Faculty of Pharmacy, University of Porto, Rua de Jorge Viterbo Ferreira 228, 4050-313 Porto, Portugal; 7 Department of Clinical Medicine, Aalborg University Hospital, Hobrovej 18-22, 9000 Aalborg, Denmark

## Abstract

Monoclonal antibodies have deserved a remarkable interest for more than 40 years as a vital tool for the treatment of various diseases. Still, there is a raising interest to develop advanced monoclonal antibody delivery systems able to tailor pharmacokinetics. Bevacizumab is a humanized immunoglobulin IgG1 used in antiangiogenic therapies due to its capacity to inhibit the interaction between vascular endothelial growth factor and its receptor. However, bevacizumab-based antiangiogenic therapy is not always effective due to poor treatment compliance associated to multiples administrations and drug resistance. In this work, we show a promising strategy of encapsulating bevacizumab to protect and deliver it, in a controlled manner, increasing the time between administrations and formulation shelf-life. Nanoencapsulation of bevacizumab represents a significant advance for selective antiangiogenic therapies since extracellular, cell surface and intracellular targets can be reached. The present study shows that bevacizumab-loaded poly (lactic-co-glycolic acid) (PLGA) nanoparticles does not impair its native-like structure after encapsulation and fully retain the bioactivity, making this nanosystem a new paradigm for the improvement of angiogenic therapy.

## Introduction

More than two centuries have passed since it began to be known the potential of monoclonal antibodies (mAbs) as unique biological tools for the treatment of several diseases, such as cancer, asthma, immune and infectious diseases^1^. Soon after the first development of mAbs in 1975, mAb-based therapy is continuing at a remarkable pace, with approximately 50 monoclonal antibodies approved by Food and Drug Administration (FDA)^2^ up to date.

Bevacizumab is a humanized IgG1 monoclonal antibody that binds to human vascular endothelial growth factor A (VEGF-A) and inhibits the interaction between VEGF-A and VEGF receptor tyrosine kinases. VEGF-A is a key regulator of tumor angiogenesis and has a noteworthy role in the promotion of growth vascular endothelial cells, regulation of vascular permeability and vasodilatation, and induction of endothelial migration^3^. Therefore, bevacizumab binds to VEGF-A and avoids the interaction between VEGF-A and their receptors, blocking of angiogenesis.

Bevacizumab may be used in the treatment of age-related macular degeneration^4^, retinal neovascularization^5^, and several cancers^6, 7^. Although antiangiogenic therapy is producing an undeniable clinical benefit for a wide range of diseases, in some cases this therapy is not beneficial due to resistance to the treatment^8^. Occasionally, the tumors manifest adaptive resistance against angiogenesis inhibitors, including bevacizumab^9^. Revascularization caused by upregulation of pro-angiogenic signals, increased tumor cells proliferation and the protection of tumors vasculature are some of the mechanisms responsible for adaptive resistance. Additionally, some studies have reported the existence of an intracellular pool of VEGF in cancer cells^10^.

Other limitations of mAb-based therapy are due to its limiting pharmacokinetics and low tumor penetration based on mAb size, tumor pressure gradient and “binding site barrier effect”^11^. Other medical applications of bevacizumab include the treatment of inflammatory and angiogenic diseases of the eye^12^. This treatment implies multiples administrations by intraocular route since intravitreal half-time of bevacizumab is small (7–10 days)^13^. Thus, patient compliance to the treatment can be compromised due to the possible onset of complications (e.g. retinal detachment, endophthalmitis, vitreous hemorrhage)^14^. Therefore, encapsulation of bevacizumab into nanocarriers holds the potential to overcome some of these drawbacks, increasing the half-time of bevacizumab, reducing the administration’s number and improving patients convenience^5, 15–18^. Additionally, NP may be targeted to specific cells and deliver the mAb inside the cell, when intracellular components are the target^10^. This may be reached with the use of poly (lactic-co-glycolic acid) (PLGA) as polymer in the NP formulation. PLGA is a synthetic polymer approved by US Food and Drug Administration (FDA) and European Medicine Agency (EMA) to polymeric nanoparticles production due to their harmless properties^19^. Controlled release, biocompatibility, biodegradability properties of PLGA NP promotes an overall decrease in toxicity and a potency enhancement of the therapy with mAbs^19, 20^. Nanoencapsulation in polymeric NP also allows increased of shelf-life of formulations, improving the long-term storage conditions and bringing fewer costs for the manufacturer and the hospital. Lyophilization of PLGA nanoparticles is a common way to increase shelf-life of formulations and reduce instability of the bevacizumab-loaded NP upon storage^21^. This method may also avoid the aggregation of PLGA NP in solution consequent of the hydrolytically unstable structure of polymer^22^.

However, methods to achieve NP formulation usually involve conditions such as the use of organic solvents, aggressive pH, high pressure and temperature, sonication, and ionic strength^23^ that may lead to structural instability of the mAb, compromising the mAb bioactivity and increasing immunogenicity^24^. Therefore, it is critical to analyse how the nanoencapsulation influences the stability of mAbs during the development of mAb-loaded PLGA nanoparticles.

The main objective of the present study was to develop a formulation of bevacizumab-loaded PLGA NP and evaluate how nanoencapsulation may affect the bevacizumab release, structure and their bioactivity. Additionally, lyophilization of nanoparticles formulation was made to improve the stability of PLGA NP.

## Materials and Methods

For the NP production, poly(lactic-co-glycolic acid), PLGA 5004 A (50:50), was gently offered by Corbion-Purac Biomaterials (Holland, Netherlands). Ethyl acetate (EA) and poly (vinyl alcohol) (PVA) was purchased from Sigma-Aldrich (St. Louis, MO, U.S.A.). The monoclonal antibody used was Bevacizumab (Avastin), which was kindly provided by Genetech Inc., South San Francisco, CA. Bevacizumab was stored at 4 °C and was provided with 51 mM sodium phosphate pH 6.2, 60 mg/mL trehalose dihydrate and 0.04% polysorbate 20. For the HPLC analysis, Trifluoroacetic acid (TFA) from Acros Organics (Morris Plains, NJ, USA) and acetonitrile HPLC Gradient Grade from Fischer Scientific (Lough-borough, UK) were used. Ultrapure water was prepared in-house with a conductivity of 0.055 μS/cm and a resistivity of 18.2 MΩ.cm, using Milli-Q station from Millipore Corp. (Madrid, Spain).

For the *in vitro* release study, sodium phosphate dibasic dehydrate, sodium phosphate monobasic monohydrate, sodium carbonate, sodium bicarbonate, were purchased from Merck (Darmstad, Germany). Bradford Protein Assay Kit from Thermo Scientific (Wilmington, DE, U.S.A.) was used in bevacizumab quantification for *in vitro* release study. For the cell culture, Human umbilical vein endothelial cells (HUVEC) were purchased from ScienCell (Carlsbad, CA, U.S.A.). Several reagents to cell culture were acquired from Sigma, such as M199 medium, Fetal Bovine Serum (FBS), heparin sodium, gelatin solution 2%, endothelial cell growth supplement (ECGS), 3-(4,5-dimethylthiazol-2-yl)-2,5-diphenyltetrazolium bromide (MTT) and dimethyl sulfoxide (DMSO). Recombinant human VEGF_165_ was purchased from BioLegend (San Diego, U.S.A.). For the BrdU incorporation assay, it was acquired Cell Proliferation ELISA, BrdU (colorimetric) Kit (Roche Applied Science, Indianapolis, IN).

### Preparation of PLGA nanoparticles

Bevacizumab-loaded PLGA NP were prepared through a modified solvent emulsification-evaporation method based on a w/o/w double emulsion technique^21, 25^. One hundred milligrams of PLGA 50:50 were dissolved in 1 mL of ethyl acetate. Then, 80 μL of a 25 mg/mL bevacizumab solution (Avastin) were added, and the polymeric solution was homogenized for 30 s using a Vibra-Cell™ ultrasonic processor with 70% of amplitude. After this homogenization, 4 mL of the surfactant solution, 2% of PVA in deionized water, were added into the primary emulsion formed (w/o) and mixed using the same sonication conditions. Finally, the second emulsion (w/o/w) was added into 7.5 mL of the same surfactant solution. This emulsion was left in a fume hood under magnetic stirring at 300 rpm during 3 h for EA evaporation. The obtained NP formulation were washed three times with ultrapure water by centrifugation. The centrifugation was performed at 40000 g for 30 min, using Beckman Avanti J26 XPI ultracentrifuge from Beckman Coulter (Brea, CA, U.S.A.). In the third centrifugation, the NP formulation was redispersed in water before storage at desired concentration. The same procedure was made to produce unloaded NP.

### Bevacizumab association efficiency (AE) and drug loading (DL)

The AE and DL were calculated by direct and indirect method. Bevacizumab quantification by direct method was made by addition of ethyl acetate to disintegrate PLGA nanoparticles. Then, a liquid-liquid extraction was performed in order to quantify bevacizumab in the aqueous phase. The AE was determined indirectly, where the amount of bevacizumab encapsulated into PLGA NP was calculated by the difference between the total amount of bevacizumab used in the NP formulation and the free bevacizumab in the supernatant isolated by ultracentrifugation in a Beckman Avanti J26 XPI ultracentrifuge at 40000 g during 30 min at 4 °C. The DL was calculated taking into account the total dry weight of PLGA NP. Briefly, AE and DL were determined using the following equations:

1 $${\rm{AE}}( \% )=\,\frac{{\rm{Total}}\,{\rm{amount}}\,{\rm{of}}\,{\rm{bevacizumab}}-{\rm{Free}}\,{\rm{bevacizumab}}\,{\rm{in}}\,{\rm{supernatant}}}{{\rm{Total}}\,{\rm{amount}}\,{\rm{of}}\,{\rm{mAb}}}\times 100$$

2 $${\rm{DL}}( \% )=\frac{{\rm{Total}}\,{\rm{amount}}\,{\rm{of}}\,{\rm{bevacizumab}}-{\rm{Free}}\,{\rm{bevacizumab}}\,{\rm{in}}\,{\rm{supernatant}}}{{\rm{Total}}\,{\rm{dry}}\,{\rm{weight}}\,{\rm{of}}\,{\rm{nanoparticles}}}\times 100$$

For the quantification of free bevacizumab in the supernatant, a high-performance liquid chromatography (HPLC) method was used. Measurements were performed using a Shimadzu UFLC Prominence System equipped with two pumps LC-20AD, an autosampler SIL-20AC, an oven CTO-20AC, a degasser DGU-20A_5_, a system controller CBM-20A and a LC solution version 1.24 SP_1_. The fluorescence detector was a Shimadzu RF-10AXL, and the column used was a Zorbax SB 300-C8 Narrow with 5μm particle size, 2.1 mm internal diameter × 260 mm length from Agilent Technologies (Santa Clara, CA, U.S.A.). Chromatographic analysis was performed in a gradient mode consisted of 0.1% TFA in ultrapure water (eluent A) and 0.1% TFA in acetonitrile (eluent B). The eluent flow rate was 1.0 ml/min and the gradient started at 35% of eluent B, held for 1 min. The gradient was increased to 45% in 0.1 min and kept constant from 1.1 to 6 min. Then, eluent B was decreased to origin value (35%) in 0.1 min and kept constant from 6.1 to 14 min. The temperature of the column was maintained at 75 °C, and the injection volume was 2 μL. Detection was realized by fluorescence, with excitation at 280 nm and emission at 360 nm. All samples were run in triplicate, and the total area of the peak was used to quantify bevacizumab.

### Freeze-drying of nanoparticles

NP were washed three times with ultrapure water, and after the last ultracentrifugation, NP were redispersed in ultrapure water before lyophilization. A set of three replicates of unloaded NP and bevacizumab-loaded NP were poured into semi-stoppered glass vials with slotted rubber closures at a maximal formulation volume of 2 mL. The samples were frozen at −80 °C for 6 h followed by lyophilization using a Modulyo 4 K freeze-dryer from Edwards (Crawley, West Sussex, U.K.) at 0.09 mbar for 72 h. The condenser surface temperature was maintained at −60 ± 5 °C. The lyophilized samples were used in ATR-FTIR spectroscopy.

### Freeze-dried samples reconstitution

In order to characterize the freeze-dried NP, the samples were reconstituted with ultrapure water at desired concentration. After 10 min of NP in contact with ultrapure water, samples were lightly shaken in a Vortex Mixer ZX Classic (Velp Scientifica) during 3 min to complete the homogenization of samples.

### Particle size and potential zeta analysis

After production and lyophilization, both formulations, in suspension and after reconstitution, were characterized for their average particle size, polydispersity index (PDI) and average zeta potential, by dynamic light scattering, using a Malvern Zetasizer Nano ZS instrument (Malvern Instruments Ltd). For the measurement, a set of three replicates were diluted with ultrapure water to an appropriate concentration.

### Characterization of nanoparticle morphology

The morphology of NP in suspension and after reconstitution (either unloaded or loaded) was observed by transmission electron microscopy (TEM) and scanning electron microscopy (SEM). Both formulations were washed by ultracentrifugation (using the same conditions described above) to eliminate the surfactant (PVA). The samples, for TEM measurements, were fixed in a grid, treated with uranyl acetate and then, were observed in a JEOL JEM-1400 electron microscope (JEOL Ltd, Tokyo, Japan). For SEM measurements, samples were seen in a FEI Quanta 400 FEG SEM microscope (FEI, Hillsboro, OR, USA). Before the observation, samples were mounted onto metal stubs and vacuum-coated with a layer of gold/palladium.

### Bevacizumab *in vitro* release study

Bevacizumab *in vitro* study was performed using the bevacizumab-loaded NP in suspension. In a first step, NP were washed by ultracentrifugation (using the same conditions described above) to eliminate the non-encapsulated bevacizumab into PLGA NPs. Bevacizumab-loaded NP were dispersed in 15.0 mL of buffers with different pHs, incubated at 37 °C under magnetic stirring at 100 rpm. It was made a triplicate procedure for each pH. Aliquots (0.5 mL) were taken at predetermined time points (1, 2, 4, 8, 24, 48, 72 and 168 h) and the samples were replaced with a fresh medium maintained the same temperature. With the purpose to calculate the released bevacizumab, each aliquot was ultracentrifuged, and the released amount of bevacizumab in the supernatant was calculated by Bradford protein kit assay kit. The chosen pHs were pH 6, 7.4 and 10.

### ATR-FTIR analysis

The secondary structure of entrapped bevacizumab into PLGA NP was evaluated by Fourier Transform Infrared Spectroscopy (FTIR). The spectra analyses were realized in ABB MB3000 FTIR spectrometer from ABB (Zurich, Switzerland) equipped with a MIRacle single reflection attenuated total reflectance (ATR) accessory from PIKE Technologies (Madison, WI, U.S.A.).

Spectra of freeze-dried unloaded NP, freeze-dried bevacizumab-loaded NP, native bevacizumab, and thermally denatured bevacizumab were obtained. The spectrum of native bevacizumab was accessed by 25 mg/mL bevacizumab solution (Avastin) provided gently from Genentech. The spectrum of thermally denatured bevacizumab was obtained when a 25 mg/mL bevacizumab solution was submitted to 100 °C, to induce the denaturation of mAb. The spectra collection of all samples were made with 256 scans and a 4 cm^−1^ resolution in the region of 600–4000 cm^−1^, and a set of three replicates of each formulation was analyzed. To obtain a bevacizumab spectrum a double subtraction procedure (buffer or unloaded NP and water vapor)^26^ was made, followed by a 13 points Savitsky-Golay second derivative, and a baseline correction using 3–4 point adjustment at the amide I region (1600–1700 cm^−1^). This spectral treatment was executed using the HorizonMB FTIR software from ABB (Zurich, Switzerland). Lastly, all spectra were area-normalized for comparison using Origin 8 software (OriginLab Corporation, Northampton, MA, USA).

### Spectral Similarity analysis

Area overlap (AO) and spectral correlation coefficient (SCC) were the algorithms chosen to quantitatively compare the similarity of the FTIR spectra between bevacizumab-loaded NP and native bevacizumab and thermally denatured bevacizumab^25^. Both algorithms were used taking into account the area normalized second-derivative amide I spectra of native bevacizumab. All the results are displayed in percentage, and the percentage is correlated with the similarity of spectra. The reference chosen was the bevacizumab solution because it is known that the stability of a solid protein formulation increases with the rise of similarity to the FTIR spectra in solution^27^.

### CD analysis

With the purpose of evaluating the secondary structural content of the native bevacizumab, released bevacizumab and the thermally denatured bevacizumab, CD analysis was performed. The measurements were carried out performed using a Jasco J815 CD Spectrophotometer (Jasco Incorporated, Easton, U.S.A.) and the lamp housing was purged with nitrogen. The spectra were obtained from 200 to 260 nm using a 0.1 cm cell, a bandwidth of 1 mm, a data pitch of 0.5 nm, a data integration time of 2 sec, and a scanning speed of 50 nm/min. The sample volume required was 0.4 mL. All spectra were the average of 8 scans. CD spectrum of the buffer was subtracted from the sample spectrum and smoothed using 9 Savitzky-Golay points before conversion to absolute CD values. Native bevacizumab, released bevacizumab from PLGA NP and thermally denatured bevacizumab were analyzed in triplicate. Native bevacizumab at 0.150 mg/mL was used as a reference. To obtain the spectrum of thermally denatured bevacizumab, a bevacizumab solution at 0.150 mg/mL was submitted to 90 °C, in order to induce the denaturation of mAb.

### Fluorescence spectroscopy

Fluorescence excitation and emission spectra of released bevacizumab and native bevacizumab were compared with a view to observing structural conformational changes of tertiary structure. The measurements were carried out using a Felix fluorescence RTC 2000 spectrometer (Photon Technology International, Canada, Inc. 347 Consortium Court London, Ontario N6E 2S8), using a 75-W Zenon arc lamp coupled to a monochromator. The fluorescence excitation spectra were obtained from 200 to 300 nm, fixing the emission excitation wavelength at 337 nm. The fluorescence emission spectra were obtained from 300 to 530 nm with an integration time of 0.1 s and 1 nm of step size, fixing the excitation wavelength at 280 nm. All spectra were collected with using an excitation slit width of 0.25 mm and an emission slit width of 6 mm. The buffer spectrum was subtracted from the sample spectrum and normalized based on bevacizumab concentration. Native bevacizumab at 0.075 mg/mL at pH 10 was used as a reference. The spectrum of thermally denatured bevacizumab was assessed when a bevacizumab solution at 0.075 mg/mL at pH 10 was warmed up from 25 °C to 90 °C, using a heating rate of 1 °C/min, to induce the denaturation of mAb.

### Cell cultures

HUVEC cells were cultured in M199 medium, supplemented with 20% of FBS, 0.1 mg/mL of heparin and 0.03 mg/mL of endothelial cell growth supplement. HUVECs were harvested on 1% w/v gelatin-coated tissue culture. The medium was renewed every 2 to 3 days until cells reached confluency. HUVEC monocultures were seeded at a density of 3 × 10^3^ cells/cm^2^ for all conditions tested.

In some experiments, HUVEC cells were treated with 50 ng/ml of VEGF. Different concentrations of Bevacizumab, Bevacizumab-loaded NP or unloaded NP, were tested.

### MTT cell toxicity assay

HUVEC monocultures were seeded at a density of 3 × 10^3^ cells/cm^2^ without heparin and ECGS and incubated during 24 hours. Then, HUVEC monocultures were incubated for 24, 48 or 72 hours, with 50 ng/mL of VEGF and 0,1; 1; 10 and 100 µg/mL of Bevacizumab (Avastin), Bevacizumab-loaded NP, or unloaded NP. After the incubation period, the media was removed, and 150 µL of 0.5 mg/mL MTT solution was added at each well, followed by incubation of the plates at 37 °C during 4 h. The reaction was terminated by removal of the media and addition of 150 µL of dimethyl sulfoxide. The levels of reduced MTT were determined by measuring the absorbance at 590 nm and 630 nm using a microtiter plate reader (Biotek-Synergy H1 Hibrid reader, using the Gen5 2.01 program). The % of MTT reduction was calculated as follows:

3 $$ \% \,{\rm{MTT}}\,{\rm{reduction}}=\frac{{\rm{absorbance}}\,{\rm{of}}\,{\rm{treated}}\,{\rm{cells}}}{{\rm{absorbance}}\,{\rm{of}}\,{\rm{untreated}}\,{\rm{control}}\,({\rm{cells}}\,{\rm{culture}}\,{\rm{without}}\,{\rm{VEGF}})}\times 100\,$$

The final results of MTT reduction, are expressed as the percentage of the absorbance of control cells (cells culture without VEGF).

### Bromodeoxyuridine (BrdU) proliferation assay

The BrdU cell proliferation assay is an immunoassay for the quantification of BrdU, which is incorporated into newly synthesized DNA during the proliferative period of the cells. HUVEC cells were cultured in 96-well plates at a density of 3 × 10^3^ cells/cm^2^ and treated with 50 ng/mL of VEGF and 10 µg/mL of Bevacizumab, Bevacizumab-loaded NP, or Unloaded NP. BrdU labeling reagent (final concentration, 10 mM) was added after 48 h of culture. At 72 h, the culture media was removed, the cells were fixed, and the DNA was denatured in one step by adding FixDenat. Next, the cells were incubated with the anti-BrdU-POD antibody for 90 minutes at room temperature. After the removal of the antibody conjugate, the cells were washed, and the substrate solution was added. The reaction product was quantified 15 min after, by measuring the absorbance using a microtiter plate reader (Biotek-Synergy H1 Hibrid reader, using the Gen5 2.01 program) at 370 nm with a reference wavelength of 492 nm. The % of BrdU incorporation was calculated as follows:

4 $$ \% \,{\rm{BrdU}}\,{\rm{incorporation}}=\frac{{\rm{absorbance}}\,{\rm{of}}\,{\rm{treated}}\,{\rm{cells}}}{{\rm{absorbance}}\,{\rm{of}}\,{\rm{untreated}}\,{\rm{control}}\,({\rm{cells}}\,{\rm{culture}}\,{\rm{without}}\,{\rm{VEGF}})}\times 100\,$$

### Statistical Analysis

For the performed statistical analysis, the GraphPad Prism Software vs. 6.0 (GraphPad Software Inc.) was used. Differences between groups were compared using one-way analysis of variance (ANOVA) Tukey post hoc test. Results are expressed as a mean ± standard deviation from a minimum of three independent experiments. Differences were considered significant at *p < 0.05, **p < 0.01, ***p < 0.001, ****p < 0.0001.

## Results

### Nanoparticles characterization

Modified solvent emulsification-evaporation method based on a w/o/w double emulsion technique was the optimized method used to produce successfully, unloaded NP and bevacizumab-loaded NP^21, 25, 28^. Both formulations were characterized regarding mean particle size, PDI, zeta potential through Zetasizer. Bevacizumab AE and DL were determined by fluorescence spectroscopy associated with HPLC (Table 1). There was no siginificant differences in the determination of bevacizumab AE and DL by direct and indirect method (data not shown). The mean particle size increased up to 198.6 ± 5.4 nm and PDI increased up to 0.160 ± 0.033 when bevacizumab was encapsulated into PLGA NP. However, the negative surface charge decreased to −20.8 ± 1.4 for bevacizumab-loaded NP. To avoid physical and chemical instability of nanoparticles in a colloidal suspension, lyophilization is a conventional method used. Thus, in Table 1 is also described the physicochemical properties of unloaded NP and bevacizumab-loaded NP after lyophilization with no cryoprotectant added. An increase in mean particle size and PDI and a decrease in potential zeta was observed for both freeze-dried NP.

**Table 1:** Physicochemical properties of unloaded NP and bevacizumab-loaded NP before and after freeze-drying.

	Unloaded NP		Bevacizumab-loaded NP	
	Before freeze-drying	After freeze-drying	Before freeze-drying	After freeze-drying
Particle size (nm)	168.4 ± 5.4	259.4 ± 7.0	198.6 ± 5.4	299.8 ± 2.2
Polydispersity index (PDI)	0.104 ± 0.003	0.262 ± 0.031	0.160 ± 0.033	0.412 ± 0.028
Zeta Potential (mV)	−23.1 ± 1.5	−20.6 ± 0.4	−20.8 ± 1.4	−16.6 ± 1.1
Association efficiency (%)	ND	ND	82.47 ± 0.56	ND
Drug Loading (%)	ND	ND	1.62 ± 0.01	ND

Values are expressed as a mean ± standard deviation. ND: not determined.

**Figure 1:**
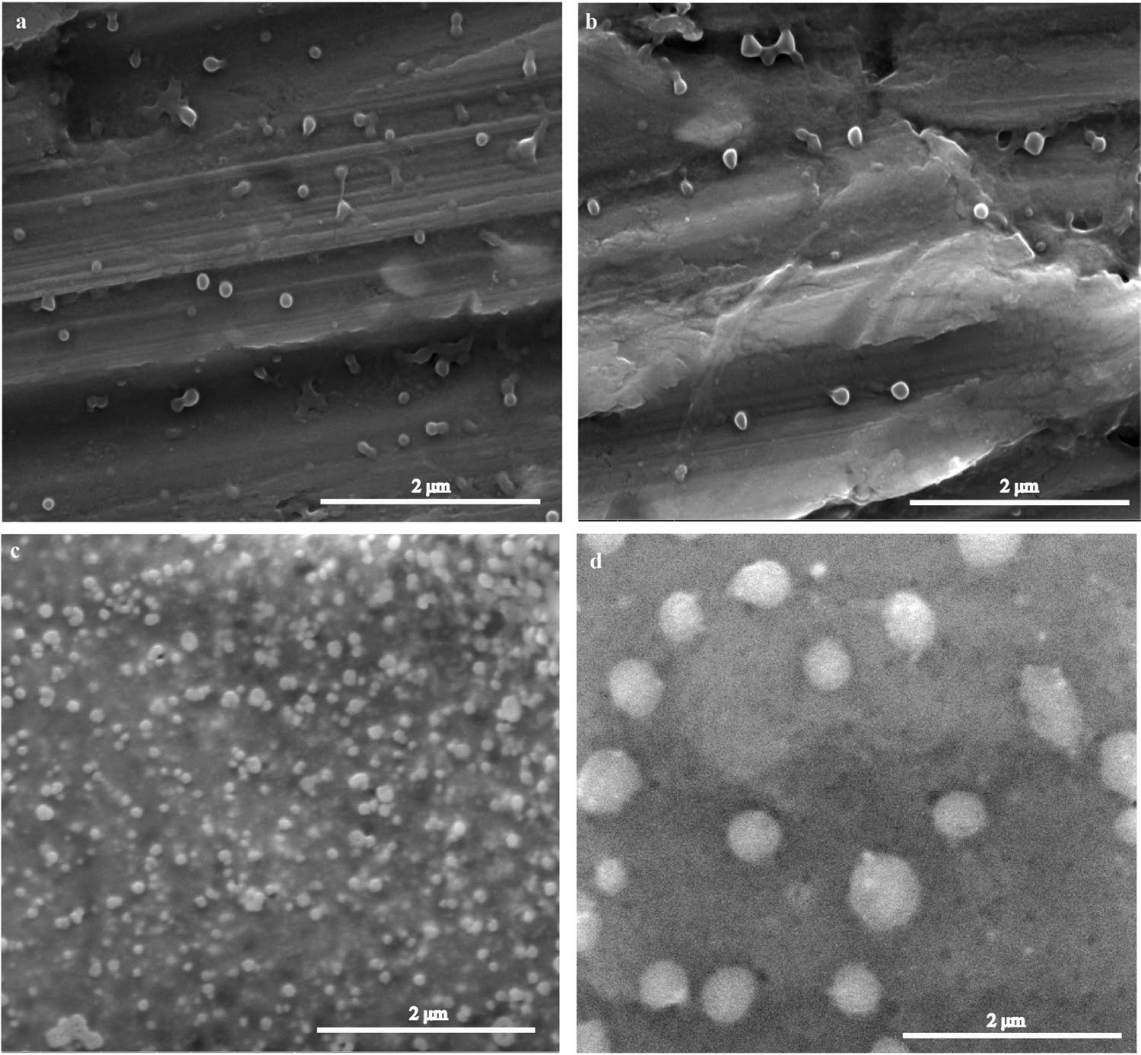
SEM microphotographs of unloaded NP (**A**), bevacizumab-loaded NP after production (**B**), freeze-dried unloaded NP (**C**) and freeze-dried bevacizumab-loaded NP (**D**). Scale bar: 2 μm.

**Figure 2:**
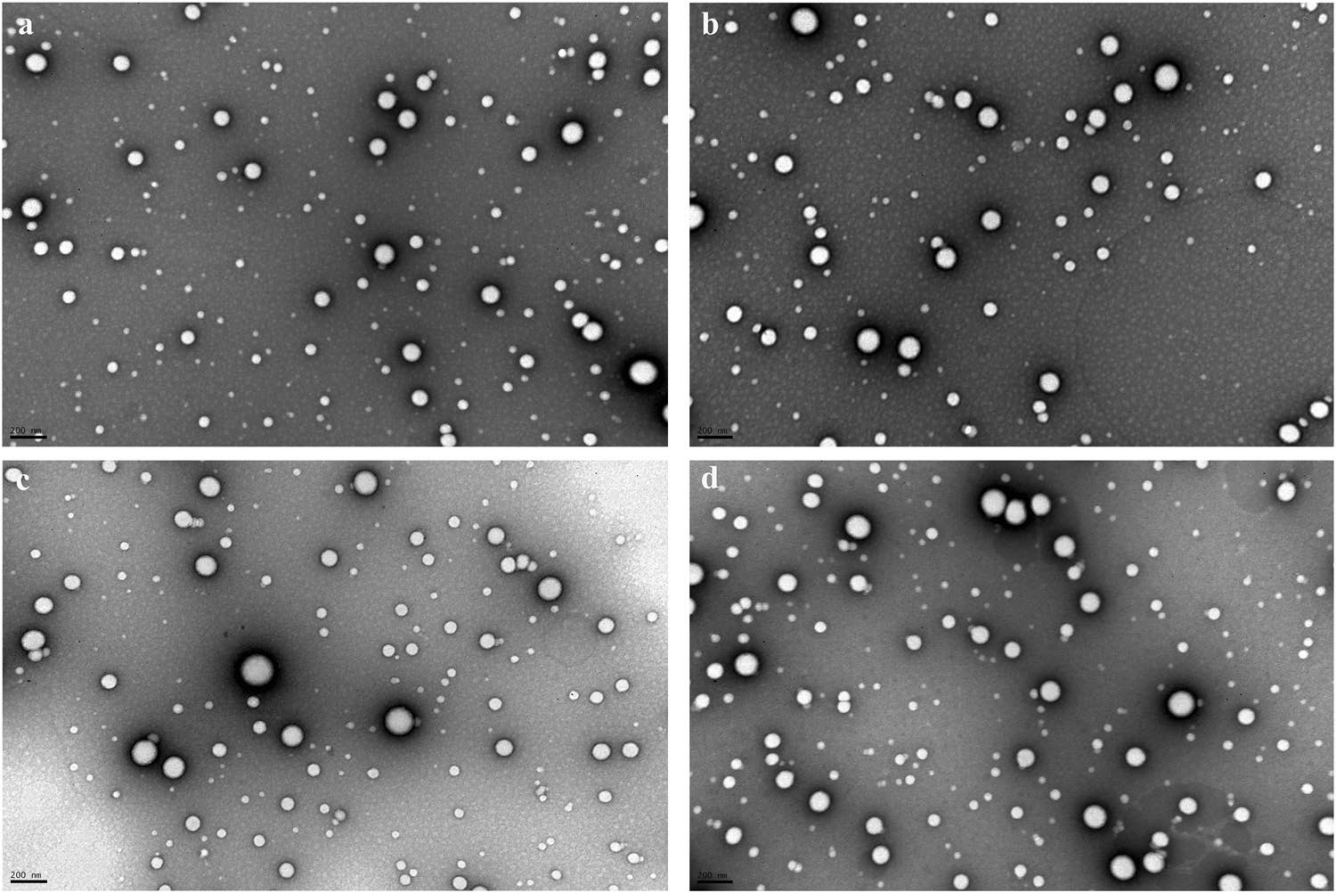
TEM microphotographs of unloaded NP (**A**), bevacizumab-loaded NP after production (**B**), freeze-dried unloaded NP (**C**) and freeze-dried bevacizumab-loaded NP (**D**). Scale bar: 200 nm.

The visualization of the morphology of unloaded NP and bevacizumab-loaded NP has been assessed through TEM and SEM. SEM provides information about the surface of nanoparticles, whereas TEM gives information about the shape of nanoparticles. The removal of PVA from NP formulations is mandatory, to obtain the best microscopic appearance of nanoparticles. Unloaded NP and bevacizumab-loaded NP were observed after formulation and after their lyophilization with no cryoprotectant added by SEM (Fig. 1) and by TEM (Fig. 2). The results obtained either TEM or SEM showed that all formulations had similar characteristics with a spherical shape and smooth surface. Additionally, it is also observed some nanoparticle aggregation and a bigger size for the resuspended freeze-dried samples comparatively to fresh NP.

**Figure 3:**
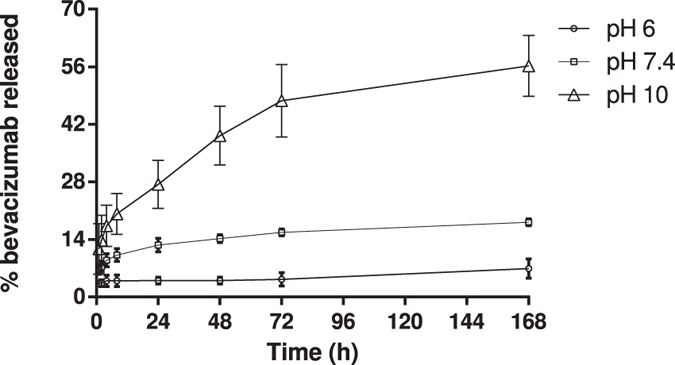
*In vitro* pH-dependent bevacizumab release profile from PLGA NP at pH 6 (circle), pH 7.4 (square), pH 10 (triangle) (n = 3, bars represent SD).

To explain the kinetics of bevacizumab release from PLGA NP, the pH-dependent release profile of bevacizumab-loaded NP was tested at 3 differents pHs (6, 7.4 and 10). In Fig. 3, it is shown the *in vitro* pH-dependent bevacizumab release profile from PLGA NP during 168 hours after formulation. The percentage of released bevacizumab increased with increasing of pH, demonstrating that PLGA NP exhibit a pH-dependent release profile.

### Bevacizumab structure after encapsulation

**Figure 4:**
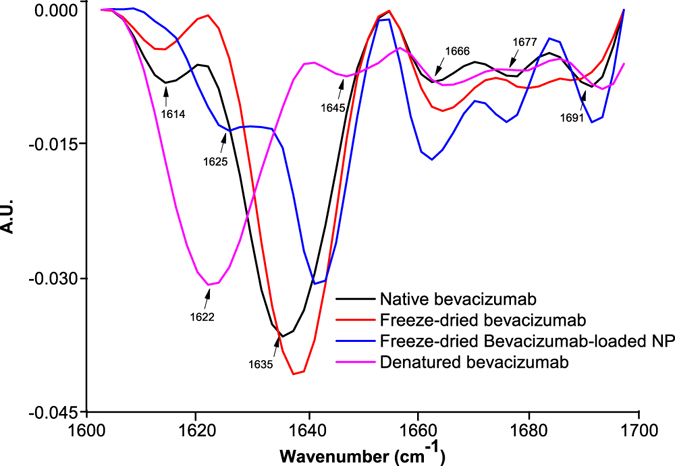
Second derivative amide I FTIR spectra of native bevacizumab, freeze-dried bevacizumab, freeze-dried bevacizumab-loaded NP and thermally denatured bevacizumab.

**Figure 5:**
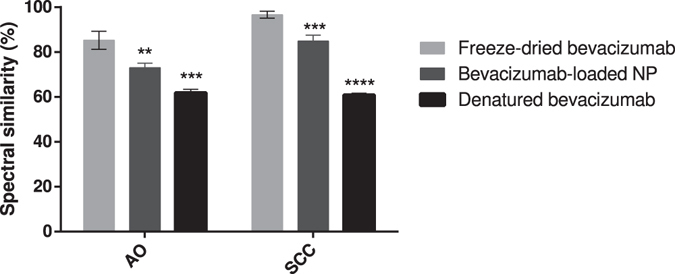
Area overlap (AO) and spectral correlation coefficient (SCC) percentages of freeze-dried bevacizumab, bevacizumab-loaded NP and thermally denatured bevacizumab. Results are significantly different (**p < 0.01, ****p < 0.0001) from native bevacizumab.

For the determination of putative structural changes of bevacizumab inside PLGA NP, ATR-FTIR spectroscopy was performed. Thus, to see the changes of bevacizumab structure, spectrum of bevacizumab-loaded NP was compared with native and thermally denatured bevacizumab. Qualitative (Fig. 4) and quantitative (Fig. 5) changes were obtained by area normalized second derivative and area overlap (AO) and spectral correlation coefficient (SCC), respectively. To get the spectra of bevacizumab-loaded NP, only freeze-dried NP was used. Actually, to assess the protein secondary structure in an aqueous medium, it is it is desirable that protein concentrations be higher than 3 mg/mL^29^. For that reason, solid samples were taking into account for this technique.

The second derivative spectrum of native bevacizumab showed that bevacizumab secondary structure is composed by 80.7% of β-sheet (1614 cm^−1^, 1635 cm^−1^, 1691 cm^−1^) and 19.7% of β-turns (1662 cm^−1^,1677 cm^−1^). Changes in bevacizumab secondary structure reveal when these bands suffer modifications, either in a shift or decrease/increase in intensity. Figure 4 shows that, when native bevacizumab was freeze-dried, the main band characteristic of the β-sheet content (1935 cm^−1^) increased and occurred a right shift of 2 or 3 cm^−1^, for the wavenumber 1635, 1662 and 1677 cm^−1^. Additionally, the spectrum of bevacizumab-loaded NP showed some modifications in comparison with native and lyophilized bevacizumab. In fact, there was the onset of a wavenumber at around 1625 cm^−1^, the disappearance of the band at 1614 cm^−1^, decrease of the band at 1641 cm^−1^, and increase the bands 1666 and 1677 cm^−1^.

AO indicates the quantitative modifications of bevacizumab secondary structure, whereas SCC represents the changes in the band positions^25^. After NP production, bevacizumab structure was preserved in about 73.0 ± 2.0, meaning that suffered a structural change of about 27.0%. Additionally, results also demonstrated that the own lyophilization process caused changes structural of about 14.7% and only 61.8 ± 2.0% of bevacizumab structure was maintained in denatured bevacizumab.

### Bevacizumab structure after release

**Figure 6:**
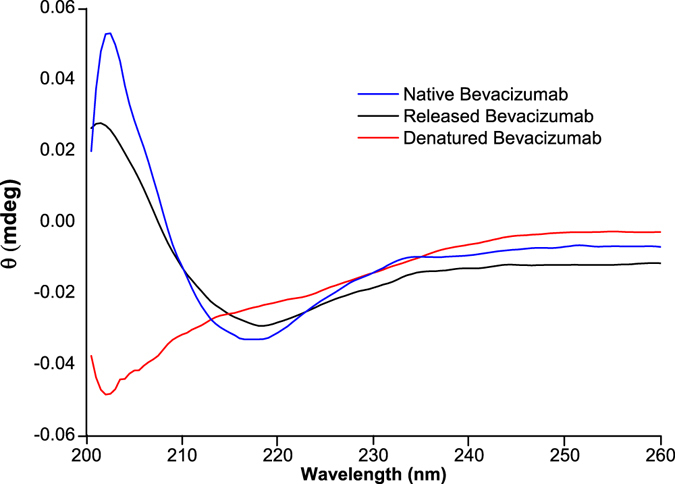
Far-UV spectra of native bevacizumab, released bevacizumab and thermally denatured bevacizumab.

The secondary structure of bevacizumab after release from PLGA NP was analyzed by CD spectroscopy. CD spectra of native, thermally denatured and released bevacizumab were obtained and compared. The CD spectrum of 0.1 mg/mL bevacizumab solution at pH 10 was used as a reference to native bevacizumab structure. Figure 6 shows that secondary structure of native bevacizumab is dominated by β-sheet structure because the spectrum showed a minimum at about 218 nm and a maximum at 202 nm. Although the minimum wavelength keeps with release bevacizumab, CD spectrum of thermally denatured bevacizumab was totally different.

**Figure 7:**
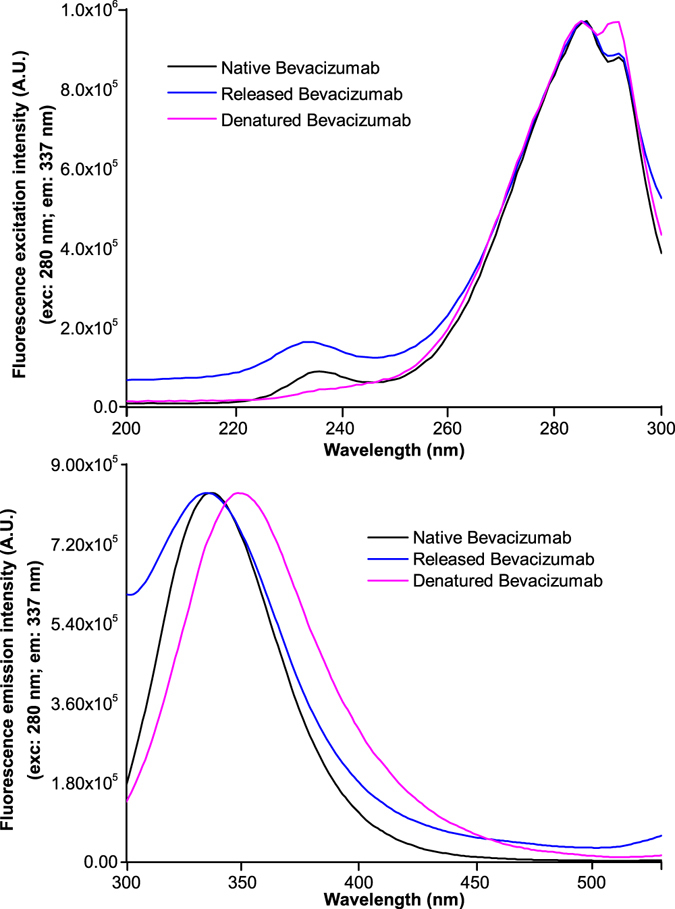
Fluorescence excitation (**A**) and emission spectra (**B**) of native, released and thermally denatured bevacizumab. Fluorescence excitation was fixed at 280 nm and fluorescence emission at 337 nm.

Fluorescence spectroscopy was the technique used to evaluate the conformational changes in the bevacizumab structure after its release. The fluorescence spectra of native, released and thermally denatured bevacizumab are shown in Fig. 7. The spectrum of native bevacizumab at pH 10, which was used as a reference, shows a maximum of excitation and emission fluorescence intensities at around 285 and 336 nm, respectively. The same results are achieved from release bevacizumab. However, it was observed that fluorescence emission spectrum of thermally denatured bevacizumab has been shifted from 336 to 349 nm.

### Bevacizumab bioactivity

**Figure 8:**
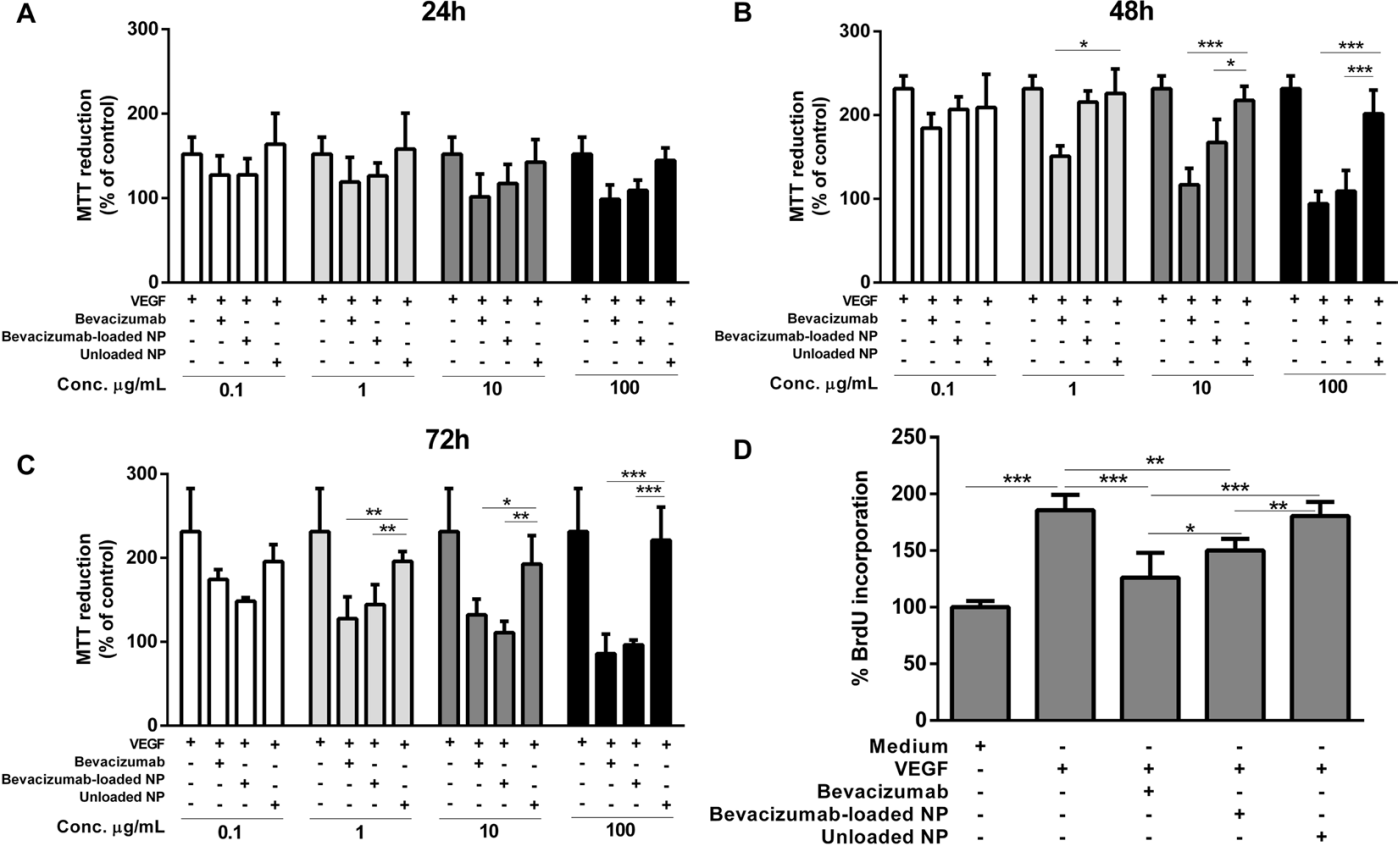
Effect of native bevacizumab (Avastin), bevacizumab-loaded NP and unloaded NP on cell viability and proliferation of HUVEC cells. (**A**–**C**) MTT assay of HUVEC cells treated with 50 ng/mL of VEGF and different concentrations (0,1; 1; 10 and 100 µg/ml) of Bevacizumab, Bevacizumab-loaded NPs, or Unloaded NPs at different time points (24 h (**A**); 48 h (**B**) and 72 h (**C**)). (**D**) BrdU incorporation assay of HUVEC cells treated with 50 ng/mL VEGF and 10 µg/ml of Bevacizumab, Bevacizumab-loaded NPs, or Unloaded NPs for 72 h. In all graphs, bars represent mean values ± SD (n = 5–6 values per condition). One representative experiment out of three total is shown. *P < 0.05; **P < 0.01; and ***P < 0.001, determined as described in materials and methods.

Bioactivity in HUVEC was tested using two different proliferation assays, MTT and BrdU. The proliferation rate of HUVEC cells was evaluated by MTT assay at 24, 48 and 72 hours. For all time-points, native bevacizumab, bevacizumab-loaded NP, and unloaded NP were added to HUVEC cells. Cell were treated with 50 ng/mL of VEGF and four different concentrations were tested taking into account the concentration of native bevacizumab. After 24 hours, no significant differences were found in all samples (Fig. 8A). After 48 hours, for lower concentrations (0.1 and 1 μg/mL), there were not found significant differences between unloaded NP and bevacizumab-loaded NP (Fig. 8B). However, for the higher concentrations (10 and 100 μg/mL), significant differences between unloaded NP and native bevacizumab and bevacizumab-loaded NP were discovered. In fact, there were no significant differences detected between native bevacizumab and bevacizumab-loaded NP. When we performed the assay after 72 hours assay, we observed a significant increase in the differences between unloaded NP and native bevacizumab and bevacizumab-loaded NP. Interestingly, we observed that the 1 μg/mL concentration the bevacizumab-loaded NP start to have an effect. (Fig. 8C).

Additionally, we also quantify the the percentage of BrdU incorporation (Fig. 8D) in HUVEC cells. Based on the MTT results, we selected the 10 μg/mLconcentration. The addition of VEGF 50 ng/mL alone caused an increase of 54% of BrDU incorporation compared to the control group (without VEGF). Results showed that HUVEC proliferation had been significantly decreased for the native bevacizumab and bevacizumab-loaded NP groups. As expected the cell proliferation was not affected by unloaded NP. However, the HUVEC proliferation was significantly different for the bevacizumab and bevacizumab-loaded NP.

## Discussion

PLGA nanoparticles are produced aiming at a controlled release of bevacizumab, aiming the increase of shelf-life, half-life and structural stability of the encapsulated mAb. After unloaded NP production, PLGA NP showed a small mean particle size (168.4 ± 5.4 nm), a little PDI (0.104 ± 0.003) and a negative charge of −23.1 ± 1.5 mV (Table 1). The negative charge of PLGA NP is due to its deprotonated carboxylic end groups of lactic and glycolic acid, and its value is a good indicator that NP are stable^30^. However, after encapsulation of bevacizumab into PLGA NP, the mean particle size increased more 1.5 fold than unloaded NP. These results may be explained by the high size of bevacizumab (149 kDa and 21.4 nm of radius minimum) and a high adsorption of bevacizumab on the particle surface^31^. Additionally, upon encapsulation, the negative charge of loaded nanoparticles decreased up 0.89 times. This reduction is due to the positive charge that bevacizumab displays at pH 7.4, since its isoelectric point is 8.3^32^. Despite the decrease, the formulation is stable because it is described that a minimum zeta potential of ± 20 mV is desirable^33^. Bevacizumab was successfully encapsulated with an AE of 82.47 ± 0.56% and a DL of 1.62 ± 0.01%.

To avoid the aggregation of NP, fusion of NP and the hydrolysis of the PLGA,both formulations were freeze-dried without any cryoprotectant. The increase in particle size and PDI and the decrease of potential zeta obtained from both freeze-dried NP are indicative of some aggregation and fusion of nanoparticles^34^. Indeed, it was demonstrated that during the freeze-drying process in the freezing step, it is possible to occur aggregation and occasionally irreversible fusion of NP, if any cryoprotectant is added to the formulation^25^. Thus, it is important to add cryoprotectant to nanoparticles formulation before the freeze-drying process.

Nanoparticles should be spherical and have a smooth surface to be less influenced by shear and allow better interaction with cell surface enhancing cellular uptake. Actually, all the formulations in our study shown have these characteristics. Additionally, SEM and TEM results are in agreeing with DLS results, with regard to size and nanoparticle aggregation of freeze-dried NP.

*In vitro* bevacizumab release showed for each pH a controlled release until the 168 h. Results also revealed that release profile for bevacizumab from PLGA NP is pH-dependent, where the amount of bevacizumab released increased with increasing pH. However, the release rate of bevacizumab-loaded from PLGA NP is lower than expected. This tendency of the low release profile of mAbs is due to several factors, such as the degradation of the polymer, diffusion of the mAb from the PLGA NP, size of mAb, isoelectric point of mAb and nanoparticle porosity^35^. Bevacizumab has an isoelectric point of 8.3, meaning that above this value, bevacizumab is negatively charged. As PLGA NP have a negative charge, at pH lower than 8.3, there is a strong electrostatic attraction between bevacizumab and NP, decreasing the release rate. Furthermore, as bevacizumab has a large size, its diffusion transport from NP is limited, being the release rate widely associated with the polymer degradation. It is known that polymer degradation is influenced by several factors, involving mechanisms of hydrolysis, enzymatic cleavage, physicochemical characteristics of the polymer and physicochemical parameters^36^. For instance, PLGA is degraded strongly at the alkaline and acidic conditions due to the ester hydrolysis caused by acid-base catalysis. In fact, our results show that at pH 10, the amount of bevacizumab released was greater. However, the low amount of bevacizumab released at pH 6 and 7.4 may also be explained knowing that this *in vitro* study does not account for the cellular complexity involved in the degradation process. The selection of the pH range was based on the normal physiology and disease condition of the human body as well as to prove the AE of bevacizumab-loaded NP. Thus, pH 6 was chosen once the cellular uptake of nanoparticles is intended. Indeed, the internalization of NP by endocytosis often use the endosomal-lysosomal pathway^37^. Thus, this pH was selected since the pH of endosomes/lysosomes is around 5.5–6.5^38^. Conversely, taking into account the biological relevance of this study, the physiological pH 7.4 was selected. Despite pH 10 has not biological significance, it was chosen to prove the association efficiency of bevacizumab-loaded NP, showing the influence interactions between bevacizumab and PLGA NP. In addition to the well-described non-covalent interactions between mAb and polymeric nanoparticles (e.g. ionic and hydrophobic interactions), some covalent interactions may occur^39^. For instance, *Chiu et al*. demonstrated the effect of multivalent liposomal therapeutic antibody constructs in the modulation of malignant cell survival pathways due to the extensive cross-linking of target/antibody complex^40^. Indeed, some authors have proved the reduction in drug release from PLGA microparticles due to the surface crosslinking of PLGA microparticles^41^. On the other hand, *Mordenti et al*. also demonstrated a slow release of a recombinant human mAb from PLGA microspheres in order to treat ocular disease by intravitreal route^42^. Summarily, bevacizumab release from PLGA NP showed a slow and pH-dependent release profile, which means that the present formulation allows for a reduction of the frequency of administrations, reducing the cost of therapy. However, *in vivo* studies should be made to prove that PLGA NP allow a slow release of bevacizumab.

ATR-FTIR spectroscopy is one of the most powerful non-invasive techniques to assess the secondary structure of proteins inside NP^26^. The secondary structure of mAb can be determined by analysis of the amide I region (1700–1600 cm^−1^) of the spectrum, corresponding to the C = O stretching vibrations of the amide group, bending of the N-H bond and stretching of the C-N bonds. The C = O stretching vibrations of the ester group of PLGA arise at around 1750 cm^−1^ and therefore, the interpretation of secondary structure is made without any interference. Structural modifications can be monitored through a comparison of the area normalized second-derivative amide I spectrum of native bevacizumab and the intended sample. As described above, bevacizumab is dominated by β-sheet, which is characteristic of immunoglobulins IgG^43^. When bevacizumab was lyophilized, it was observed a right shift of these bands, which is in agreeing with literature. Lyophilization process still allows the formation of intermolecular β-sheets due to water removal, causing an increase in β-sheet content and a decrease of a α-helix^44^.

Through the analysis of bevacizumab-loaded NP spectrum, the appearance of the band at 1625 cm^−1^ is attributed to the formation of intermolecular β-sheet caused by aggregation phenomena^45^. In fact, thermally denatured bevacizumab spectrum have two additional bands at 1622 and 1645 cm^−1^ related with intermolecular β-sheet and unordered structure, respectively^46^. However, significantly differences between the spectrum of thermally denatured bevacizumab and bevacizumab-loaded NP were found, meaning that bevacizumab was not completely denatured (Fig. 5).

Although there were bevacizumab conformational changes while encapsulated into PLGA NP, a refolding of bevacizumab structure can occur after its release. Thus, to evaluate the conformational modifications of bevacizumab secondary structure and tertiary structure, CD and Fluorescence spectroscopy were used. As mAbs are optically active macromolecules absorbing circular polarized light, CD spectroscopy can be used to determine its secondary structural content^47^. Thus, the absorption of peptide bond varies according to the types of secondary structure of protein. However, this technique does not allow the determination of bevacizumab-loaded NP due to the light scattering caused by PLGA nanoparticles. Thus, CD spectroscopy was used to determined the secondary structural content of native, thermally denatured and released bevacizumab (Fig. 6).

The spectrum of released bevacizumab did not show relevant shifts of the characteristic spectrum of native bevacizumab, indicating the prevalent β-sheet structure of bevacizumab. The decrease of the ellipticity signal may be attributed to a small variability in the rearrangement of bevacizumab structure. The spectrum of thermally denatured bevacizumab shows that bevacizumab structure is dominated by random coil structure with a minimum at about 202 nm. Several authors associate the appearance of random coil structures in a protein with protein denaturation^48, 49^. Actually, *Vermeer et al*. showed that with the increase of temperature, structural modifications of an IgG occur being translated by a decrease of β-sheets and β-turns structures and an increment of α-helix and random coil structures^50^.

Monitoring of conformational changes of bevacizumab tertiary structure by fluorescence spectroscopy is crucial since the secondary structure can be intact even when the tertiary structure is modified or lost^51^. The maximum fluorescence emission intensity observed for native bevacizumab was 336 nm, which is in agreement with others results previously reported^52^. Released bevacizumab spectrum was practically similar to the native bevacizumab spectrum, concluding that the solvent accessibility of tryptophan residues is maintained after its release. Structural changes can be analyzed by a decrease in the maximum fluorescence emission intensity and/or by a shift of maximum wavelength^51^. Red-shift from 336 to 349 nm of maximum fluorescence emission was observed to thermally denatured bevacizumab. This shift indicates that the tryptophan moiety is more solvent exposed, due to temperature-induced conformational changes^51^. Briefly, our results showed that the secondary and tertiary structure of bevacizumab was maintained after its release from NP.

Several authors studied the inhibitory effects of bevacizumab on angiogenesis using cultured HUVEC *in vitro* ^53, 54^. Thus, in our experience, the biological activity of bevacizumab was determined by its capacity to inhibit the HUVEC proliferation induced by VEGF_165_. VEGF_165_ was chosen because is the main isoform of VEGF-A that presents the most physiological relevant activity^54, 55^. For that reason, 50 ng/mL of VEGF was added at all the formulations^56^. Typically, the formulations represented as bevacizumab and bevacizumab-loaded NP showed time and concentration-dependent viability in HUVEC cells (Fig. 8). For the lower concentration (0.1 μg/mL) was not found significant differences due to the low concentration of bevacizumab (100 ng/mL) compared to the VEGF concentration (50 ng/mL). Figure 8B,C shows that there were no significant differences between bevacizumab and bevacizumab-loaded NP, proving that bioactivity of bevacizumab was maintenance even when it was encapsulated into NP. MTT results also showed that PLGA NP do not present cytotoxicity for HUVEC cells, which is in agreement of the literature^28^.

The BrdU proliferation assay was realized to investigate the bevacizumab effect in HUVEC proliferation at the level of the DNA (Fig. 8D). As expected, the BrdU incorporation was 1.85-fold higher after the addition of 50 ng/mL VEGF_165_ in the medium of HUVEC cells. Additionally, no significant differences were found between unloaded NP and the medium with VEGF showing there was no interference in the normal proliferation of HUVEC cells. A significant decrease in HUVEC proliferation was also observed when cells were treated with bevacizumab and bevacizumab-loaded NP, being the results in concordance with MTT assay. However, the decline in HUVEC proliferation caused by bevacizumab was significantly different from bevacizumab-loaded NP. This different may be attributed to the slow release of bevacizumab from PLGA NP since BrdU assay was performed only during 72 hours. To overcome this problem, a higher concentration of bevacizumab-loaded NP and a higher incubation time could be the solution.

Shortly, our experimental results showed that bevacizumab bioactivity was not changed with the nanoencapsulation process. Although the secondary structure of bevacizumab was changed when encapsulated into PLGA NP, our results by CD and fluorescence spectroscopy showed bevacizumab refolding upon release from PLGA NP. This refolding process was confirmed by bioactivity *in vitro* studies, where the bioactivity of bevacizumab was kept, demonstrating the success of bevacizumab encapsulation. Despite the slow release of bevacizumab-loaded NP observed with *in vitro* release study, biological *in vitro* study showed that bevacizumab can be released and acts in the same way than bevacizumab. Our study represents a new paradigm for antiangiogenic therapy since bevacizumab is protected and released in a controlled manner, increasing the time between administrations. However, in oder to envision the clinical application of bevacizumab-loaded NPs, *in vitro* and *in vivo* studies about nanoparticles cellular uptake, the mechanisms underlying bevacizumab delivery at the sub-cellular level are certainly needed. Nevertheless, these results are promising as a novel strategy to improve antiangiogenic-based therapies.
